# Does neighborhood fast-food outlet exposure amplify inequalities in diet and obesity? A cross-sectional study^1^,^2^

**DOI:** 10.3945/ajcn.115.128132

**Published:** 2016-05-11

**Authors:** Thomas Burgoine, Nita G Forouhi, Simon J Griffin, Søren Brage, Nicholas J Wareham, Pablo Monsivais

**Affiliations:** 3 UKCRC Centre for Diet and Activity Research (CEDAR) and Medical Research Council Epidemiology Unit, University of Cambridge School of Clinical Medicine, Institute of Metabolic Science, Cambridge, United Kingdom; and; 4 Department of Public Health and Primary Care, Institute of Public Health, University of Cambridge, Cambridge, United Kingdom

**Keywords:** deprivation amplification, educational attainment, fast-food, geographic information systems, obesity

## Abstract

**Background:** Greater exposures to fast-food outlets and lower levels of education are independently associated with less healthy diets and obesity. Little is known about the interplay between these environmental and individual factors.

**Objective:** The purpose of this study was to test whether observed differences in fast-food consumption and obesity by fast-food outlet exposure are moderated by educational attainment.

**Design:** In a population-based cohort of 5958 adults aged 29–62 y in Cambridgeshire, United Kingdom, we used educational attainment–stratified regression models to estimate the food-frequency questionnaire–derived consumption of energy-dense “fast foods” (g/d) typically sold in fast-food restaurants and measured body mass index (BMI; in kg/m^2^) across geographic information system–derived home and work fast-food exposure quartiles. We used logistic regression to estimate the odds of obesity (BMI ≥30) and calculated relative excess risk due to interaction (RERI) on an additive scale. Participant data were collected during 2005–2013 and analyzed in 2015.

**Results:** Greater fast-food consumption, BMI, and odds of obesity were associated with greater fast-food outlet exposure and a lower educational level. Fast-food consumption and BMI were significantly different across education groups at all levels of fast-food outlet exposure (*P* < 0.05). High fast-food outlet exposure amplified differences in fast-food consumption across levels of education. The relation between fast-food outlet exposure and obesity was only significant among those who were least educated (OR: 2.05; 95% CI: 1.08, 3.87; RERI = 0.88), which suggested a positive additive interaction between education and fast-food outlet exposure.

**Conclusion:** These findings suggest that efforts to improve diets and health through neighborhood-level fast-food outlet regulation might be effective across socioeconomic groups and may serve to reduce observed socioeconomic inequalities in diet and obesity.

## INTRODUCTION

Obesity is a risk factor for multiple chronic diseases, including type 2 diabetes, cardiovascular disease, and some cancers. Nearly two-thirds of adults are now overweight or obese in Great Britain, with the prevalence predicted to increase further by 2050 (1). These trends in obesity may be due in part to increased fast-food (or “takeaway food”) consumption. Great Britain has seen a 29% increase in financial expenditure on fast foods over the past decade (2), and £28 billion worth of fast foods are now purchased annually (2).

Foods consumed outside the home are typically less healthy than those consumed at home (3). Fast foods (e.g., pizza, burgers, and fried chicken) tend to be high in saturated fat and salt, energy dense, nutrient poor (4), and served in large portions (5). The regular consumption of meals from fast-food outlets (6) and the increasing fast-food meal consumption frequency over time have been associated with adult weight gain (7). Increased patronage of fast-food outlets has also been associated with excess weight gain over time (8).

The consumption of fast foods appears to be influenced by both individual- and neighborhood-level factors. One individual-level factor is socioeconomic status (SES),^5^ characterized in terms of income, occupation, or educational attainment (9). Adults with lower levels of education, in particular, have been reported to consume unhealthy fast foods more frequently (10, 11) and to visit fast-food outlets more often than those with higher levels of education (12). These education-related differences in consumption have been implicated in the higher amounts of adiposity generally found in less-educated groups (13–15).

Neighborhood food environments, including those beyond the residential neighborhood, may also be a cue for higher intakes of fast foods. Although the evidence base is mixed and methodologically heterogeneous (16), neighborhood exposure to fast-food outlets has been associated with the purchasing (17) and consumption (18–21) of fast foods. Positive associations between exposure to fast-food outlets and body weight have also been observed (18, 20, 22–26). For both fast-food consumption and body weight, nonhome environmental exposures may be particularly influential, especially in the neighborhood surrounding the workplace (27, 28).

Although both individual-level socioeconomic and neighborhood-level drivers of diet and weight have been recognized, limited research has examined how food environment associations with food consumption and body weight might differ by SES. Public health theories, such as deprivation amplification, have long sought to understand whether, how, and why unhealthy neighborhood environment exposures are more important for populations of low SES (29). However, to date, empirical evidence in support of this notion has been lacking, which constitutes an important gap in knowledge. The purpose of this study was to develop a more refined understanding of how educational attainment might serve to modify previously observed (18) main associations of fast-food consumption and measured body weight with respect to combined home and work neighborhood fast-food outlet exposure in Cambridgeshire, United Kingdom.

## METHODS

### Study sample

The Fenland Study is an ongoing population-based cohort study in adults aged 29–62 y (born between 1950 and 1975) enrolled in general practices in Cambridgeshire, United Kingdom (www.mrc-epid.cam.ac.uk/research/studies/fenland-study/). Cambridgeshire is a county in the east of England that comprises urban, suburban, and rural areas and the major cities of Cambridge and Peterborough. Recruitment for this study was conducted by the University of Cambridge Medical Research Council Epidemiology Unit from 2005 and is ongoing. At the time of the data request for these analyses, data were available for 10,452 participants. Participants completed a general lifestyle questionnaire, which included questions on highest educational attainment. Participants also completed a semiquantitative food-frequency questionnaire (FFQ) to assess the habitual consumption of foods; weight and height were measured by trained researchers. Eligibility criteria for this analysis were as follows: having complete demographic, anthropometric, socioeconomic, and FFQ data and information on home and work address and being employed. The analytic sample was therefore restricted to 6123 participants (see participant flow diagram in **Supplemental Figure 1**). The participant data used in this study were collected between 2005 and 2013 and analyzed in 2015. All of the study procedures were approved by the Health Research Authority National Research Ethics Service Committee East of England–Cambridge Central. The Fenland Study volunteers provided written informed consent.

### Exposure: home and work fast-food outlets

Data on food outlet locations were sourced from local councils throughout the study area in December 2011. The accuracy of such data from local councils was shown previously (30). Fast-food outlets were classified as shown in **Supplemental Table 1** (30); these are the types of food outlets to which new United Kingdom local planning restrictions are beginning to apply. Chain supermarkets were also identified as those with a substantial share of the United Kingdom grocery market (31). Food outlet locations were geocoded at the postcode level by using a geographic information system. We previously described in detail the methods used for defining food environment exposures at home and work (27). Briefly, home and work neighborhoods were delineated as 1-mile straight-line radius (circular) buffers, centered on home and work addresses. A previous study suggested that this definition of neighborhood was relevant to shopping behavior in a sample of United Kingdom adults (32). The numbers of fast-food outlets and supermarkets were summed within neighborhoods, with no denominator necessary because of the consistent size of the buffer used between participants. Counts of fast-food outlets were combined (summed) across home and work neighborhoods as the primary exposure in this analysis. To minimize residual confounding in multivariable models (18), counts of supermarkets were also combined across home and work neighborhoods for use as a covariate.

### Outcomes: dietary intake and BMI

We had 3 outcome variables. First, we estimated the consumption of energy-dense foods that can typically be obtained from a fast-food outlet (although not exclusively)—here referred to as “fast food”—using data from the FFQ. We summed the consumption in grams per day of pizza, burgers, chips (fried potatoes), fried fish, and fried chicken. Second, BMI (in kg/m^2^) was calculated from measured height and weight. Third, those with a BMI ≥30 were classified as obese.

### Statistical analysis

We used linear and logistic regression models to examine associations between educational attainment, fast-food consumption (g/d), BMI, and odds of being obese. Educational attainment groups were as follows: lowest (≤11 y of education), middle (12–13 y of education), and highest (>13 y of education). We used general linear models to estimate adjusted marginal means with 95% CIs for fast-food consumption and BMI across quartiles of combined home and work fast-food outlet exposure. We then calculated subgroup-specific estimates of fast-food consumption and BMI across education groups.

Following STROBE (Strengthening the Reporting of OBservational studies in Epidemiology) guidelines, we used logistic regression with a single reference category (least exposed, most educated) to estimate the separate and combined associations of fast-food outlet exposure and educational attainment on the odds of being obese (33). We tested for interaction on an additive scale using relative excess risk due to interaction (RERI), calculated as RERI = OR_11_ − OR_10_ − OR_01_ + 1, where ORs are odds ratios for being obese for those who are least educated and most exposed to fast-food outlets (OR_11_), those who are least educated and least exposed to fast-food outlets (OR_10_), and those who are most educated and most exposed to fast-food outlets (OR_01_) (33). RERI scores >0 suggest a positive interaction or a greater risk due to interaction than would be attributable to the additive effects of each of these factors in the absence of one another (33).

All of the models were adjusted for known confounders through the inclusion of a number of covariates: age, sex, household income (<£20,000, £20,000–£40,000, or >£40,000/y), combined home and work exposure to supermarkets, total energy intake derived from FFQ data (kJ/d; for fast-food consumption models only), physical activity energy expenditure (kJ/kg per day) (34), and smoking status (for BMI and obesity models only). Because many participants had incomplete data for physical activity energy expenditure, measured by using combined acceleration and heart rate sensors worn for up to 6 d (35), the analytic sample for BMI models was further restricted from 6123 to 5958 (Supplemental Figure 1). Our analytic sample remained representative of the full Fenland Study sample across key variables (**Supplemental Table 2**). All of the analyses were conducted by using PASW Statistics 21 (SPSS Inc.).

## RESULTS

### Sample characteristics

Descriptive statistics for the analytic sample, overall and stratified by educational attainment, are presented in **Table 1**. Fast-food consumption was, on average, 9.7 g/d (32%) higher in the least educated group than in the most educated group. BMI was, on average, 2.0 units higher in those with the lowest levels of educational attainment, and the percentage classified as obese in the middle and lowest education groups was double the number in the highest education group.

**TABLE 1:** Characteristics of participants in the Fenland Study sample (Cambridgeshire, United Kingdom)^1^

	Educational attainment^2^			
	Highest (*n* = 2033)	Middle (*n* = 2719)	Lowest (*n* = 1206)	All (*n* = 5958)
Age, y	46.5 ± 7.6	47.9 ± 7.0	48.4 ± 6.8	47.5 ± 7.2
Men, *n* (%)	1071 (52.7)	1295 (47.6)	472 (39.1)	2838 (47.6)
Energy intake,^3^ kJ/d	8088 ± 2502	8204 ± 2757	8181 ± 2959	8159 ± 2717
Physical activity energy expenditure, kJ · kg^−1^ · d^−1^	53.5 ± 20.1	54.5 ± 21.9	54.8 ± 23.5	54.2 ± 21.7
Household income >£40,000, *n* (%)	1557 (76.6)	1259 (46.3)	364 (30.2)	3180 (53.4)
Current or ex-smoker, *n* (%)	719 (35.4)	1284 (47.2)	625 (51.8)	2628 (44.1)
Owns car, *n* (%)	1822 (89.7)	2611 (96.1)	1138 (94.5)	5571 (93.6)
Food environment exposures^4^				
Combined supermarket availability	6.9 ± 5.9	4.1 ± 4.2	4.2 ± 3.9	5.1 ± 5.0
Combined fast-food outlet availability	26.0 ± 20.8	19.3 ± 17.2	21.4 ± 17.3	22.0 ± 18.7
Crude dietary and anthropometric outcomes				
Fast-food consumption, g/d	30.6 ± 25.5	35.3 ± 29.4	40.3 ± 38.4	35.1 ± 30.4
BMI, kg/m^2^	25.5 ± 4.1	27.2 ± 4.8	27.5 ± 5.0	26.7 ± 4.7
Obese (BMI ≥30), *n* (%)	250 (12.3)	634 (23.3)	295 (24.5)	1179 (19.8)
Adjusted dietary and anthropometric outcomes^5^				
Fast-food consumption,^6^ g/d				
Model 1^7^	Ref	6.2 (4.8, 7.6)**	11.2 (9.6, 13.0)**	—
Model 2^8^	Ref	3.9 (2.5, 5.5)**	8.2 (6.4, 10.2)**	—
BMI,^6^ kg/m^2^				
Model 1^9^	Ref	1.6 (1.4, 1.9)**	2.0 (1.7, 2.4)**	—
Model 2^10^	Ref	1.4 (1.1, 1.7)**	1.8 (1.4, 2.2)**	—
Obese, BMI (≥30)^11^				
Model 1^9^	Ref	2.12 (1.78, 2.52)**	2.24 (1.83, 2.73)**	—
Model 2^10^	Ref	2.03 (1.69, 2.45)**	2.13 (1.71, 2.66)**	—

1 Values are means ± SDs unless otherwise stated. *n* = 5958. **P < 0.001. Ref, reference group.

2 Educational attainment (3 groups): lowest, ≤11 y of education; middle, 12–13 y of education; and highest, >13 y of education.

3 4.18 kJ = 1 kcal.

4 Based on counts of food outlets across home and work neighborhoods.

5 Modeled by using linear and logistic regression models.

6 Values are βs; 95% CIs in parentheses.

7 Model adjusted for participant age, sex, and daily energy intake.

8 Model adjusted for age, sex, daily energy intake, household income, and supermarket and fast-food outlet exposure.

9 Model adjusted for age, sex, and smoking status.

10 Model adjusted for age, sex, smoking status, household income, physical activity energy expenditure, and supermarket and fast-food outlet exposure.

11 Values are ORs; 95% CIs in parentheses.

### Associations of fast-food consumption, BMI, and obesity with educational attainment

Greater fast-food consumption, higher BMI, and increased odds of being obese were associated with lower educational levels, with evidence of dose-response associations. Linear and logistic regression results, with adjustment for age, sex, daily energy intake (fast-food consumption model only), and smoking status (BMI and obesity models only), are shown in Table 1. Those who were least educated consumed an additional 11.3 g fast food/d (95% CI: 9.6, 13.0 g/d) relative to those who were most educated; they also had a BMI that was 2.0 units higher (95% CI: 1.7, 2.4) and were more than twice as likely to be obese (OR: 2.24; 95% CI: 1.83, 2.73) than those who were the most educated. These associations were all highly significant (*P* < 0.001). After additional adjustment for a number of covariates including environmental exposures, those who were least educated still consumed significantly more fast food (β = 8.3 g/d; 95% CI: 6.4, 10.2 g/d), had a significantly higher BMI (β = 1.8; 95% CI: 1.4, 2.2), and were more than twice as likely to be obese (OR: 2.13; 95% CI: 1.71, 2.66) than those who were most educated.

### Associations between fast-food exposure and fast-food consumption

**FIGURE 1:**
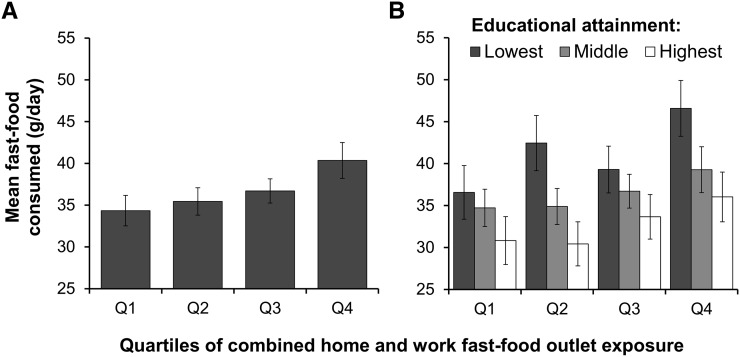
Mean (95% CI) fast-food consumption per quartile of combined home and work fast-food outlet exposure in the Fenland Study sample (*n* = 6123) (A), and stratified by educational attainment (B). Results are from a general linear model adjusted for age, sex, household income, daily energy intake, and supermarket availability. Panel A also adjusted for educational attainment. Numerical limits (counts of fast-food outlets) for each quartile of exposure: Q1 (least exposed) = 0–5, Q2 = 6–17, Q3 = 18–34, and Q4 (most exposed) = 35–96. For educational attainment: lowest, ≤11 y of education; middle, 12–13 y of education; and highest, >13 y of education. Q, quartile.

Greater fast-food consumption was associated with greater fast-food outlet exposure, with evidence of a dose-response association. **Figure 1A** shows the estimated mean fast-food consumption per quartile of combined home and work fast-food outlet exposure, with adjustment for known confounders and educational attainment. Those who were most exposed to fast-food outlets consumed 40.4 g fast food/d (95% CI: 38.2, 42.5 g/d), which was significantly more than those in any other exposure group and 6.0 g/d more than those who were least exposed.

### Analyses stratified by education

Figure 1B shows the mean fast-food consumption per quartile of fast-food outlet exposure, stratified by educational attainment. Although fast-food outlet exposure remained positively associated with fast-food consumption across all education groups, those who were most educated consumed the least at all levels of exposure. On average across all exposure levels, those who were least educated consumed 26% more fast food/d than did those who were the most educated. However, high fast-food outlet exposure did appear to further amplify differences in consumption between education groups. Moreover, those who were least educated and most exposed to fast-food outlets consumed 46.6 g/d (95% CI: 43.3, 49.9 g/d) and those who were most educated and least exposed consumed 30.8 g/d (95% CI: 28.0, 33.7 g/d).

### Associations between fast-food exposure and BMI

**FIGURE 2:**
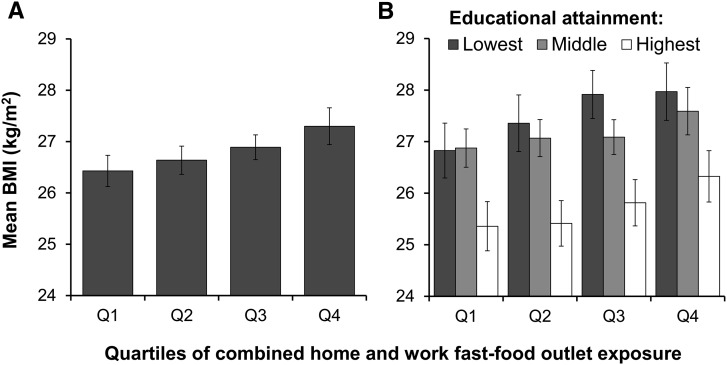
Mean (95% CI) BMI per quartile of combined home and work fast-food outlet exposure in the Fenland Study sample (*n* = 5958) (A), and stratified by educational attainment (B). Results are from a general linear model adjusted for age, sex, household income, smoking status, physical activity energy expenditure, and supermarket availability. Panel A also adjusted for educational attainment. Numerical limits (counts of fast-food outlets) for each quartile of exposure: Q1 (least exposed) = 0–5, Q2 = 6–17, Q3 = 18–34, and Q4 (most exposed) = 35–96. For educational attainment: lowest, ≤11 y of education; middle, 12–13 y of education; and highest, >13 y of education. Q, quartile.

Greater fast-food outlet exposure was associated with greater BMI, with evidence of a dose-response association. **Figure 2A** shows the mean BMI per quartile of combined home and work fast-food outlet exposure. Those who were most exposed to fast-food outlets had a significantly higher BMI (27.3; 95% CI: 26.9, 27.7) than those in any other exposure group, which was 0.9 units higher than those who were least exposed.

### Analyses stratified by education

Figure 2B shows the mean BMI per quartile of fast-food outlet exposure, stratified by educational attainment. Although fast-food outlet exposure was positively associated with body weight in all education groups, being better educated was associated with a lower BMI, and being most educated was associated with a significantly lower BMI at all exposure levels. On average across exposure levels, those who were least educated had a 1.8 unit (7%) higher BMI than those who were most educated. Differences in mean BMI between those who were least educated and most exposed (28.0; 95% CI: 27.4, 28.6) and those who were most educated and least exposed (25.4; 95% CI: 24.9, 25.9) were especially pronounced.

Increased odds of obesity (BMI ≥30) were associated with increased fast-food outlet exposure and lower educational level, with evidence of interaction on an additive scale. **Table 2** shows logistic regression results for each combination of fast-food outlet exposure quartile and educational level, with a single reference category (least exposed, most educated). The RERI was 0.88, indicating a positive additive interaction and an excess risk of obesity for those who were most exposed to fast-food outlets and least educated. Furthermore, greatest fast-food outlet exposure was not associated with a significantly increased odds of obesity for those in the middle and highest education groups (Table 2, far right column), whereas for those who were least educated, being most exposed was significantly associated with more than twice the odds of obesity (OR: 2.05; 95% CI: 1.08, 3.87). At every level of fast-food outlet exposure, lowest education was significantly associated with greater odds of obesity (Table 2, bottom row) (ORs: 2.07–2.18; all *P* < 0.05).

**TABLE 2:** Additive interaction between fast-food outlet exposure and educational attainment on the likelihood of being obese [BMI (in kg/m^2^) ≥30] modeled using logistic regression in the Fenland Study sample^1^

	Combined home and work fast-food outlet exposure								
	Q1 (0–5 outlets)		Q2 (6–17 outlets)		Q3 (18–34 outlets)		Q4 (35–96 outlets)		
	Obese/not obese, *n*	Value	Obese/not obese, *n*	Value	Obese/not obese, *n*	Value	Obese/not obese, *n*	Value	Fast-food outlet exposure (Q4) within education strata
Educational attainment^2^									
Highest	54/379	Ref	61/393	1.15 (0.77, 1.72)	61/363	1.38 (0.90, 2.10)	74/648	1.26 (0.76, 2.06)	1.03 (0.51, 2.09)
* P*				0.488^3^		0.140^3^		0.372^3^	0.926^4^
Middle	185/613	2.05 (1.46, 2.87)*	175/526	2.39 (1.70, 3.37)*	155/557	2.22 (1.55, 3.18)*	119/389	3.11 (2.00, 4.83)*	1.45 (0.92, 2.28)
* P*		<0.001^3^		<0.001^3^		<0.001^3^		<0.001^3^	0.107^4^
Lowest	70/240	1.99 (1.33, 2.98)**	65/201	2.31 (1.53, 3.50)*	91/262	2.84 (1.91, 4.24)*	69/208	3.12 (1.96, 4.98)*	2.05 (1.08, 3.87)**
* P*		0.001^3^		<0.001^3^		<0.001^3^		<0.001^3^	0.030^4^
Lowest education within fast-food outlet quartile exposure strata		2.07 (1.35, 3.17)**		2.12 (1.40, 3.22)*		2.10 (1.39, 3.18)*		2.18 (1.43, 3.32)*	
* P*		0.001^5^		<0.001^5^		<0.001^5^		<0.001^5^	

1 Values are ORs (95% CIs) adjusted for age, sex, household income, smoking status, physical activity energy expenditure, and supermarket availability; *n* = 5958. Measure of interaction on an additive scale: RERI = 0.88. RERI scores >0 suggest a positive interaction and a departure from additivity. *P < 0.001; **P < 0.05. Q, quartile; Ref, single reference group (those who were the highest educated and least exposed to fast-food outlets); RERI, relative excess risk due to interaction.

2 Educational attainment (3 groups): lowest, ≤11 y of education; middle, 12–13 y of education; and highest, >13 y of education.

3 ORs and *P* values relative to the reference group (Ref).

4 ORs and *P* values relative to those who were least exposed to fast-food outlets within strata of educational attainment.

5 ORs and *P* values relative to those who were the highest educated within strata of fast-food outlet exposure.

## DISCUSSION

In this study, with its detailed information on combined home and work environmental exposures and individual-level characteristics including measured BMI, we confirmed earlier work that showed that fast-food consumption, body weight, and the likelihood of being obese are associated with neighborhood fast-food outlet exposure (18, 22, 36) and educational attainment (10), with evidence of dose-response associations. Associations with fast-food outlet exposure were also observed in education-stratified models, with differences in fast-food consumption and BMI that were significantly different across education groups. However, highest fast-food outlet exposure appeared to further amplify differences in fast-food consumption across education groups and was significantly associated only with odds of obesity in those who were least educated. Evidence of additive interaction in odds of obesity further suggested that the adverse influence of highest exposure to fast-food outlets was exaggerated among those who were least educated.

Our results showed that although exposure to fast-food outlets was consistently associated with fast-food consumption and body weight across all education groups, there was a clear educational gradient at every level of fast-food outlet exposure. Being more highly educated was consistently associated with lower body weight and lower intakes of energy-dense fast foods, which are associated with excess weight gain over time (8). Further research is required to determine the mechanisms by which low educational attainment confers increased vulnerability to unhealthy neighborhood fast-food outlet exposures. The apparent protective effects of education may, however, be attributed to the psychosocial, behavioral, and economic resources commonly associated with higher educational attainment. A 2010 study found that fast-food outlet exposure was associated only with the frequency of fast-food outlet visits for those with higher individual-level reward sensitivity, a psychological trait hypothesized to confer a greater responsiveness to unhealthy neighborhood environment cues, and linked closely to SES (37). Other possible mechanisms include less food and nutrition knowledge (38), fewer cooking skills or inadequate cooking equipment (39), and lower income (40) among low-SES groups.

To date, there has been limited evidence of the interplay between individual SES and neighborhood exposures in relation to health and health behaviors. For example, previous research showed that in regions of the United Kingdom with greater access to green space, income-related inequalities in mortality were attenuated (41). With respect to neighborhood food environments, a US-based study found that greater exposure to fast-food outlets was associated with body weight only among low-income individuals (36). Combining our evidence of additive interaction together with the fact that fast-food outlets tend to be more prevalent in deprived United Kingdom regions (42) provides what is perhaps the first empirical confirmation of the “deprivation amplification” hypothesis in the area of diet and obesity (43). Deprivation amplification may be an important contributor to established socioeconomic gradients in diet and health in the United Kingdom, the United States, and elsewhere.

Our results are of international significance because they contribute to an emerging evidence base that suggests that government policies to regulate neighborhood fast-food outlet exposure might succeed in improving diets and health (18). Such policies include restricting the proliferation of fast-food outlets in a number of United Kingdom regions (44) and in South Los Angeles (45). Critically, our results also suggest that these policies will be effective across socioeconomic groups and potentially serve to reduce socioeconomic inequalities in diet and health. This is important because individual-level interventions that rely heavily on individual agency for their success have largely failed to reduce health inequalities (46). Principally, this failure has been attributed to such policies proving to be ineffective in groups of low SES (47), the population demographic for whom environmental level approaches may be most effective (41).

As detailed previously (18, 27), the limitations of our metric of fast-food outlet exposure (which are not unique to this study) include the following: the use of 1-mile straight-line radius (circular) buffers to represent home and work neighborhoods, which may not necessarily match the participants’ own perceptions of “neighborhood” or be congruent with actual food-shopping behaviors in this sample; the lack of information on residential history, which, if recent residential moves have been made, could lead to exposure misclassification; some temporal mismatch arising from the capture of food outlet data at only 1 time point (2011) within the period when participant data were collected (2005–2013), which is a common consideration in research of this type (16, 48); and not accounting for food outlet exposure in wider activity spaces beyond home and work domains, such as when commuting (27). In addition, we were not able to account for time spent at home and at work, which could moderate the effects of fast-food outlet exposures in these locations (49).

We used data from a semiquantitative FFQ to estimate the consumption of energy-dense fast-food and total energy intakes. The results of FFQs are representative of usual dietary intake and are commonly used in food environment research (21) but are prone to systematic error (recall bias) and are less detailed than 24-h dietary recalls or food diaries (50). Furthermore, fast foods consumed could have been purchased from non–fast-food outlets. Although we adjusted for exposure to supermarkets, where fast-food–type foods are also available, we cannot rule out residual confounding by way of access to other food outlets.

We used highest educational attainment as our indicator of SES. Other commonly used indicators include income and occupation; however, these are generally imperfectly correlated (51). We adjusted for household income in our models, but it is possible that our results are sensitive to our selection of socioeconomic indicator. Other limitations of this study include its observational, cross-sectional study design, which limits inference on causal relations. The associations observed may reflect relocation of homes or workplaces to more easily access fast foods, or the opening of fast-food outlets where there is perceived demand, rather than the effects of fast-food outlet exposure on fast-food consumption and body weight per se. Our analysis was based on a sample of the population of Cambridgeshire who were more highly educated and less ethnically diverse than the United Kingdom population as a whole. This may influence the generalizability of these findings.

This study contributes to the international scientific literature on neighborhood food environments, diet, and health by furthering our understanding of the differential effects of fast-food outlet exposure across education groups. For the first time to our knowledge, we showed the individual and combined effects of education and fast-food outlet exposure on the consumption of energy-dense fast foods, measured body weight, and odds of obesity in a large population-based sample of adults. Although exposure to fast-food outlets afffected all socioeconomic groups, those of a lower SES consumed consistently more fast foods, tended to have higher body weights, and were more likely to be obese. We also showed how the association between exposure to fast-food outlets and obesity was most pronounced for persons of lower SES and how the combination of low SES and high fast-food outlet exposure amplified the odds of obesity. Taken together, these findings may hold implications for health inequalities and therefore the development of public health policy.

## Footnotes

5 Abbreviations used: FFQ, food-frequency questionnaire; RERI, relative excess risk due to interaction; SES, socioeconomic status.
